# Structural monitoring data repair based on a long short-term memory neural network

**DOI:** 10.1038/s41598-024-60196-2

**Published:** 2024-04-30

**Authors:** Ba Panfeng, Zhu Songlin, Chai Hongyu, Liu Caiwei, Wu Pengtao, Qi Lichang

**Affiliations:** 1 School of Civil Engineering, Tianjin City Construction University, Tianjin, 300384 China; 2 School of Civil Engineering, Qingdao University of Technology, Qingdao, 266033 China; 3 Qingdao International Airport Group Co., Ltd, Qingdao, 266108 China

**Keywords:** Structural health monitoring, Data restoration, Long short-term memory neural network, Large-span structure, Support vector machines, Civil engineering, Scientific data

## Abstract

As construction technology and project management develop, structural monitoring systems become increasingly important for ensuring large-span spatial structure safety during construction and operation. However, most of the sensors and monitoring equipment in monitoring systems are poorly serviced, resulting in frequent abnormal monitoring data, which directly leads to challenges in data analysis and structural safety assessment. In this paper, a structural response recovery method based on a long short-term memory (LSTM) neural network is proposed by studying the autocorrelation of data and the spatial correlations among data at multiple measurement points. The effectiveness and robustness of the proposed method are verified using the monitored stress data for a grid structure jacking construction process, and the influence of different data loss rates on the recovery accuracy is analysed. The recovery models are compared using a support vector machine and a Multi-Layer Perception (MLP) neural network. The proposed method can effectively restore missing data; notably, the MSE index is 0.6, and the MAPE is below 15%. The data restoration method based on the LSTM neural network is more accurate than the traditional method. Finally, the repair applicability of various types of monitored data is verified using the monitoring data from Hall F of Qingdao Jiao-dong International Airport under typhoon conditions.

## Introduction

With the continuous advancement of structural design forms, building materials, construction techniques, and so on, large-span spatial structures have rapidly developed. These structures have been widely used in constructing landmark buildings such as sports arenas, terminals, and exhibition centers. However, during the construction and operation of large-span spatial structures, strength degradation and other damages, such as wind load, environmental erosion, and initial defects, are inevitably caused, resulting in safety hazards. Therefore, establishing a health monitoring system is important to ensure the reliability of spatial structures. Various types of sensors, including vibration, displacement, and strain sensors, are used in structural health monitoring to measure structural responses and accurately and effectively assess engineering structure conditions. Evaluation results depend on a large amount of reliable monitoring data, but unforeseen factors such as equipment failures, damage to data transmission systems, and power outages inevitably arise during long-term operation of the monitoring system, leading to abnormal monitoring data and data loss^1^.

Therefore, some researchers in the field of health monitoring have explored data repair issues in engineering discussions. Data imputation and prediction for missing data have been extensively studied in other domains such as statistics^2^, metrology^3^, and economics^4^. Currently, several methods are employed for data repair, including compressive sensing, linear regression, K-nearest neighbor algorithms, neural networks, and support vector regression^5^. Bao et al.^6^ proposed a machine learning-based approach to address data reconstruction in compressive sensing. By treating the computation process as a data flow, their solution for data reconstruction based on compressive sensing is formalized as a standard supervised learning task. Ye et al^7^ introduced a novel health monitoring data reconstruction method that combines wavelet multiresolution analysis with support vector machines. Comparative validation against the traditional autoregressive moving average (ARMA) method demonstrated that the wavelet-based support vector machine exhibits better effectiveness and accuracy. Zhu et al.^8^ presented a new dynamic response reconstruction method based on the multi-rate Kalman filter (MRKF). Their approach first represents the structural system using state-space equations. Subsequently, different observation equations are defined, with the choice based on the availability of specific time sensor types. This method not only directly integrates multi-type sensor data sampled at different rates but also relaxes concurrent monitoring requirements. Addressing the limitations of direct displacement measurements, such as challenges in installing monitoring devices, Zhang et al.^9^ proposed an intelligent reconstruction method that enables real-time intelligent online reconstruction of structural displacements. From the perspective of sequence data generation, Jiang et al.^10^ introduced a novel structural health monitoring method based on dynamic response reconstruction and virtual sensing. Their approach employs a sequence-to-sequence modeling framework with a soft attention mechanism.

Several scholars have proposed leveraging correlations among data from multiple sensors for data repair. Zhang et al.^11^ utilized long-term monitoring data from the Hangzhou Olympic Sports Center steel structure to introduce a point-correlation-based interpolation method for repairing missing stress data under various data loss conditions. Results indicate that linear regression interpolation error remains at 5% when the single-point correlation coefficient exceeds 0.9. The interpolation error for continuously missing data is slightly larger than that for discretely missing data, with an ideal data loss rate not exceeding 5% and a maximum threshold of 30%. Li et al.^12^ presented a data reconstruction method based on probabilistic principal component analysis. Compared to traditional principal component analysis, this method exhibits higher accuracy, particularly in cases of continuous data loss. Wei et al.^13^ investigated a correlation-based monitoring information reconstruction approach. Using stress measurements from the Shenzhen Bay Sports Center structural health monitoring system as an example, they verified the effectiveness and practicality of their proposed method. He et al.^14^ proposed a displacement reconstruction method that fuses acceleration and strain response data induced by moving loads, with a specific focus on power spectral density (PSD) extraction. Li et al.^15^ introduced a multi-task ground-penetrating radar (mGPR) paradigm to capture inter-sensor correlations and holistically reconstruct missing data from faulty sensors. In this framework, for a specific sensor, data reconstruction involves learning not only from other known data from the same sensor but also from the entire set of known measurements across other sensors. Li et al.^16^ developed an integrated framework for enhancing structural health monitoring system data using machine learning algorithms. They applied an improved non-negative matrix factorization model to reconstruct original data obtained from the Nanjing Yangtze River Tunnel structural health monitoring system. The framework detected anomalies under different conditions and employed supervised learning methods to handle anomalies identified by non-negative matrix factorization. He et al.^17^ devised a novel method for reconstructing critical location structural responses using remote sensing measurements. Their approach, based on empirical mode decomposition and derived intermittent criteria and transformation equations from finite element modeling, directly decomposed dynamic responses measured by sensors into modal responses in the time domain.

One common method for data repair is based on Bayesian probability analysis. Zhang et al.^18^ proposed the Bayesian Dynamic Regression (BDR) method to reconstruct missing data in health monitoring. They employed Kalman filters and the Expectation-Maximization (EM) algorithm to estimate state variables (regression coefficients) and parameters. The feasibility of dynamic regression BDR was verified through examples involving buildings and large-span cable-stayed bridges. Huang et al.^19^ introduced a Bayesian Compressive Sensing (CS) algorithm for reconstructing approximately sparse signals. They explored robust handling of uncertain parameters, including integrating the precision parameter for prediction error as a “nuisance” parameter and introducing continuous relaxation processes to optimize the essential hyperparameters. The algorithm’s performance was studied using compressed data from synthetic and real signals obtained from structural health monitoring systems installed on spatial framework structures and cable-stayed bridges. Compared to other state-of-the-art CS methods (including previously published Bayesian approaches), the new CS algorithm demonstrated superior robustness in reconstructing approximately sparse signals and quantifying posterior uncertainty. Sun et al.^20^ proposed a Bayesian robust tensor learning method to extract spatiotemporal features of bridge temperature-deformation fields. During the reconstruction process of temperature-induced fields using tensor learning, they simultaneously achieved missing data recovery and anomaly data cleaning. The method’s performance was validated using continuous monitoring data from an actual cable-stayed bridge.

Ren et al.^21^ presented an incremental Bayesian matrix/tensor learning scheme to effectively attribute and predict structural responses in long-term SHM. They constructed spatiotemporal tensors, performed Bayesian tensor decomposition, and extracted latent features to fill in missing data. To predict structural responses based on long-term and incomplete sensing data, an incremental learning scheme was developed to efficiently update the Bayesian temporal decomposition model. Using concrete bridge field data (including strain and temperature records) highly correlated with strain time histories, the proposed probabilistic tensor learning framework demonstrated accuracy and robustness even under significant random deletion rates, structured deletion rates, and their combinations. Wan et al.^22^ introduced a novel approach for structural health monitoring data recovery based on multi-dimensional Gaussian processes and prior Bayesian multitask learning. They validated the method’s effectiveness in reconstructing structural health monitoring data using the 600-meter-high Guangzhou Tower as an experimental platform. Chen et al.^23^ investigated the uncertainty quantification of the Distribution-Warping Function Regression (an indirect distribution-to-distribution regression method) when reconstructing missing data distributions. By transforming the warping function into vector space using function transformations, they estimated confidence intervals based on function principal component analysis and vector space self-starting regression operators. The method was validated through simulation studies and practical applications.

Deep learning^24^, as one of the fastest-growing artificial intelligence technologies, offers unique advantages over traditional data processing methods in data mining and feature extraction. Notably, it has made significant strides in the field of data recovery. Yann Le Cun et al.^24^ emphasized that deep learning enables computational models composed of multiple processing layers to learn data representations at various abstraction levels. These methods have greatly enhanced state-of-the-art levels in various domains, including speech recognition, visual object detection, and drug discovery in genomics. Deep learning achieves this by utilizing the backpropagation algorithm to uncover complex structures within large datasets, guiding machines on how to adjust their internal parameters. These parameters are used to compute representations at each layer based on the representations from the previous layer. Specifically, deep convolutional networks have revolutionized image, video, speech, and audio processing, while recurrent networks have made breakthroughs in handling sequential data such as text and speech.

Chai et al.^25^ applied deep learning to irregular and regularly missing data reconstruction. They developed a model structure based on an encoder-decoder U-Net convolutional neural network, using randomly sampled data as input and corresponding complete data as output. The training data consisted of carefully curated synthetic seismic data and real seismic data. The network was trained using mean squared error loss and the Adam optimizer, revealing feature mappings of the randomly sampled dataset. This method was successfully applied to irregularly missing data across multiple typical datasets. Wu et al.^26^ proposed a deep learning-based MR reconstruction network that serves as a unified solution for parallel MRI, obtaining k-space data from various scanning trajectories. Furthermore, deep convolutional neural network algorithms have found applications in data repair. Zhang et al.^27^ introduced a novel method for reconstructing missing information from remote sensing images. Their unified spatiotemporal spectral framework based on deep convolutional neural networks (CNNs) combined a uniform deep CNN with additional spatiotemporal spectral information. Gao et al.^28^ presented a dynamic response reconstruction method for structural health monitoring using densely connected convolutional networks. Their designed network incorporated subpixel transformation and dropout techniques, reducing computational complexity and enhancing training efficiency. The results demonstrated accurate reconstruction of both time-domain and frequency-domain signal responses, along with strong noise resistance. Guo et al.^29^ proposed a sensor fault diagnosis and signal recovery algorithm based on convolutional neural networks (CNNs) and deep convolutional generative adversarial networks. Validation using numerical models and bridge test results revealed a fault diagnosis accuracy exceeding 90%, and the repaired signals closely matched the ground truth data. Fan et al.^30^ introduced a CNN-based method for recovering vibration data in structural health monitoring, validating its effectiveness and robustness using long-term vibration data from a pedestrian bridge.

Fan et al.^31^ proposed a novel method for dynamic response reconstruction in structural health monitoring using a Dense-Net architecture. Leveraging acceleration responses from the Guangzhou New Television Tower, this approach accurately reconstructs both time-domain and frequency-domain responses, demonstrating robust noise immunity. Ni et al.^32^ introduced a new deep learning framework for data compression and reconstruction, utilizing generative adversarial networks (GANs). Notably, even with a compression ratio as low as 10% for normal data, the reconstructed results exhibit minimal error. Liu et al.^33^ presented a missing temperature data recovery method based on long short-term memory (LSTM) networks. Validation using temperature monitoring data from the Nanjing Dashengguan Yangtze River Bridge compared the proposed method against support vector machines and wavelet neural networks. The results highlight the effective recovery of missing structural temperature data, outperforming other models in terms of accuracy. Chai et al.^25^ established a model architecture based on an encoder-decoder U-Net convolutional neural network, using random sampled data as input and corresponding complete data as output. This method surpasses Fourier transform interpolation techniques in terms of performance. In addition, Liu et al.^34^ proposed a wavelet-based residual deep learning method with U-Net as the backbone for seismic data reconstruction, achieving favorable results.

Scholars have harnessed Generative Adversarial Network (GAN) algorithms for data restoration tasks. Notably, Fan et al.^35^ introduced a segmentation-based Conditional GAN (SegGAN), a powerful deep learning model designed to address pixel-to-pixel tasks. SegGAN is specifically employed for dynamic response reconstruction in structural health monitoring. Furthermore, Jiang et al.^36^ proposed a novel data-driven GAN framework for restoring missing strain responses. This network leverages residual observations and considers spatiotemporal relationships with other strain sensors. The approach was successfully implemented and validated on practical concrete bridges. Additionally, Liu et al.^37^ presented a deep convolutional GAN architecture, featuring a generator structure with an encoder-decoder and an adversarial discriminator. The proposed GAN model aims to comprehend the complete signal content and generate realistic assumptions for missing signals.

Currently, recurrent neural networks (RNN) are the primary method for repairing and reconstructing time series data in deep learning. Wang et al.^38^ proposed a three-level bidirectional RNN specifically tailored for Bridge Weigh-in-Motion (BWIM) applications. This bidirectional RNN (BRNN) incorporates both LSTM and attention mechanisms to further enhance network performance. Additional experimental data demonstrates that the BRNN outperforms traditional methods like Moving Force Identification (MFI) in estimating axle loads. In another approach, Fan et al.^39^ introduced a robust deep learning method that unifies the interpolation of missing data and damage identification within a single framework. This method leverages an autoencoder (AE) architecture based on the LSTM structure, simulating missing data using dropout mechanisms on input channels. The reconstruction error serves as both the loss function and damage indicator. The proposed approach was validated using the quasi-static response (cable tension) of a cable-stayed bridge from the First International Conference on Structural Health Monitoring (IPC-SHM). The results demonstrate effective integration of missing data imputation and damage identification within the unified framework. Furthermore, Shin et al.^40^ proposed an RNN model enhanced with external feedback to improve the accuracy and effectiveness of reconstructing dynamic responses of measured structures during sensor data reconstruction. To evaluate the performance of their method, they trained simple RNNs, LSTMs, and gated recurrent units (GRUs) using acceleration data from a laboratory-scale three-layer and six-layer shear building frame. Additionally, Chen et al.^41^ introduced a strain reconstruction method that combines nonlinear deep learning (DL) components with linear autoregressive (AR) components. Their approach utilizes Bi-GRU and CNN within the DL component to better capture long-term and short-term patterns in structural health monitoring (SHM) data, as well as the correlations between these two types of data. The method was thoroughly validated using long-term SHM data from a large-span steel box girder suspension bridge. The results indicate that the DL and AR hybrid model achieves higher accuracy in reconstructing both long-term and short-term missing data compared to CNN-based models.

Ma et al.^42^ proposed a method for detecting and repairing missing power data based on Self-Organizing Map (SOM) and LSTM neural networks. The combination of SOM and LSTM effectively addresses the challenge of handling larger data volumes and increased randomness in power consumption data. Perez-Ramirez et al.^43^ introduced a recurrent neural network (RNN) to predict the structural response of large buildings. They used acceleration time history responses from one floor as input to the neural network and responses from another floor as the output layer. The trained neural network model validates the predicted structural response to new lateral loads. Ju et al.^44^ proposed an event-related abnormal data recovery framework based on Gated Recurrent Unit (GRU) neural networks. This model achieves high accuracy in recovering abnormal data.

In summary, there is currently limited research on missing data in structural health monitoring systems, and existing research focuses primarily on the repair of single monitoring data such as stress or vibration. However, actual engineering monitoring data can be classified into multiple types, such as acceleration, displacement, and stress. Therefore, in this paper, deep learning technology is introduced and a missing data repair method based on a LSTM for various types of monitoring data is proposed. The autocorrelation of single measurement points and the cross-correlation between multiple measurement points are considered. The highest correlated structural response is used as the input data for the LSTM model, and the response of the missing segment is used as the output for data repair.

## Structural health data recovery based on LSTM

**Figure 1:**
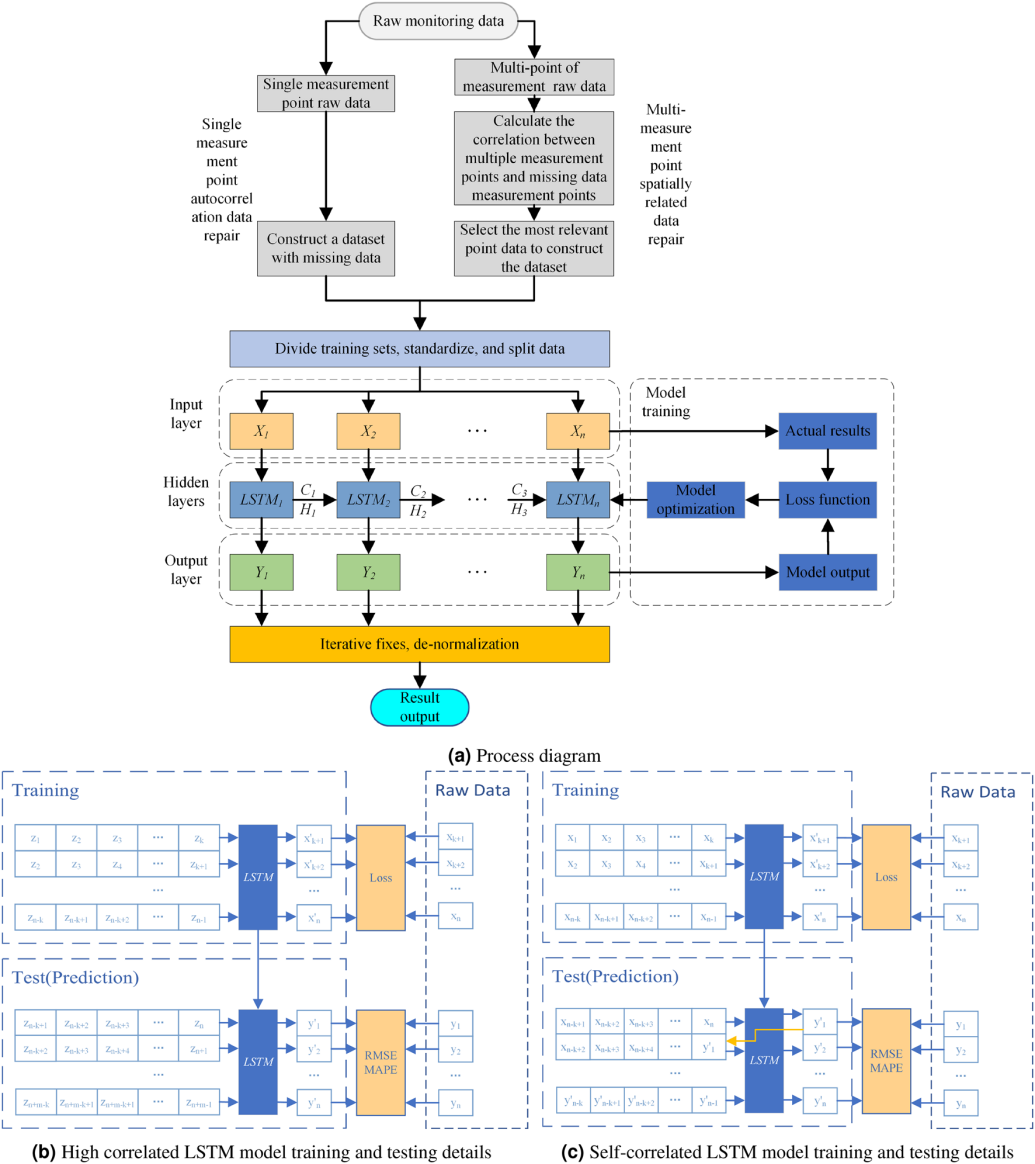
Structure monitoring data repair method based on LSTM neural network.

There are many types of missing data during the operation of the SHM system. Missing data can be categorized as single point missing or multiple point missing according to the number of measuring points, or they can be categorized as long sequence data missing or short sequence data missing according to the amount of missing data. To address the diverse data loss issues faced by structural monitoring systems, intelligent techniques are required for efficient and accurate data repair, which aims to ensure data integrity. In this paper, data repair methods are proposed for different types of data loss in structural monitoring systems by considering deep learning methods and correlation analysis. Specifically, two approaches are proposed: (1) For isolated data loss at a single measurement point, the self-correlation of the data is considered. The LSTM model is employed to learn the data features from the existing monitoring data through data mining, and then the trained model is used to predict and repair the missing data segments to restore the structural response. (2) For data loss in multiple measurement points with spatial correlation, the data from strongly correlated measurement points are selected and used as a data set for training the LSTM model. Similarly to the approach for single-point self-correlation loss, the trained model is used to predict and repair the missing data. The specific process is illustrated in Fig. 1.

The specific steps are as follows:

1. If there are multiple related adjacent measuring points near the reconstruction target measuring point, the high cor-relation repair method can be used. Otherwise, the self-correlation repair method can be used.
2. When there are adjacent measurement points to the reconstruction target measurement point, the data of the measurement points are subjected to correlation analysis, and the measurement point data with the strongest correlation is selected for high correlation repair. When there are no adjacent measurement points related to the reconstruction target measurement point, the data reconstruction is performed using the autocorrelation repair method.
3. Construct a dataset, train an LSTM neural network, and reconstruct the target measurement data.
  - High correlation repair methods
     $\{ {x_1},{x_2},{x_3}, \cdots ,{x_n},{y_1},{y_2}, \cdots {y_m}\}$ is the original data time series for reconstruction target, where $\{ {x_1},{x_2}, \cdots ,{x_n}\}$ is the assumed complete data, $\{ {y_1},{y_2}, \cdots {y_m}\}$ is the original data of the assumed missing part, and $\{ {z_1},{z_2},{z_3}, \cdots ,{z_n},{z_{n + 1}},$ ${z_{n + 2}}, \cdots {z_{n + m}}\}$ is the original data time series with high correlation. These are used to construct the training set, validation set and test set used in autocorrelation repair. The training set and validation set input matrix is $$\left[ {\begin{array}{*{20}{c}}{{z_1}}&{}{{z_2}}&{} \cdots &{}{{z_k}}\\ {{z_2}}&{}{{z_3}}&{} \cdots &{}{{z_{k + 1}}}\\ \cdots &{} \cdots &{} \cdots &{} \cdots \\ {{z_{n - k}}}&{}{{z_{n - k + 1}}}&{} \cdots &{}{{z_{n - 1}}} \end{array}} \right] $$, the training set and validation set output target is column vector ${[{x_{k + 1}},{x_{k + 2}}, \cdots {x_{k + n - 1}}]^T}$, the test set input matrix is $$\left[ {\begin{array}{*{20}{c}} {{z_{n - k + 1}}}&{}{{z_{n - k + 2}}}&{} \cdots &{}{{z_n}}\\ {{z_{n - k + 2}}}&{}{{z_{n - k + 3}}}&{} \cdots &{}{{z_{n + 1}}}\\ \cdots &{} \cdots &{} \cdots &{} \cdots \\ {{z_{n + m - k}}}&{}{{z_{n + m - k + 1}}}&{} \cdots &{}{{z_{n + m - 1}}} \end{array}} \right] $$, and the test set predicted output is column vector ${[y{'_1},y{'_2}, \cdots y{'_m}]^T}$. The LSTM neural network model is trained using the training set and validation set, that is, the model is trained to predict the next time step data of the reconstruction target data time series using the available data of k time steps in the highly correlated data time series. After training is completed, the test set input matrix is input into the LSTM neural network to obtain the predicted column vector ${[y{'_1},y{'_2}, \cdots y{'_m}]^T}$.
  - Self-correlation repair method
    $\{ {x_1},{x_2},{x_3}, \cdots ,{x_n},{y_1},{y_2}, \cdots {y_m}\}$ is the original data time series, where $\{ {x_1},{x_2}, \cdots ,{x_n}\}$ is the assumed complete data, $\{ {y_1},{y_2}, \cdots {y_m}\}$ is the original data of the assumed missing part. These are used to construct the training set and validation set and test set used in autocorrelation repair. The training set and validation set input matrix is $$\left[ {\begin{array}{*{20}{c}} {{x_1}}&{}{{x_2}}&{} \cdots &{}{{x_k}}\\ {{x_2}}&{}{{x_3}}&{} \cdots &{}{{x_{k + 1}}}\\ \cdots &{} \cdots &{} \cdots &{} \cdots \\ {{x_{n - k}}}&{}{{x_{n - k + 1}}}&{} \cdots &{}{{x_{n - 1}}} \end{array}} \right] $$, the training set and validation set output target is column vector ${[{x_{k + 1}},{x_{k + 2}}, \cdots {x_{k + n - 1}}]^T}$, the test set input matrix is $$\left[ {\begin{array}{*{20}{c}} {{x_{n - k + 1}}}&{}{{x_{n - k + 2}}}&{} \cdots &{}{{x_n}}\\ {{x_{n - k + 2}}}&{}{{x_{n - k + 3}}}&{} \cdots &{}{y{'_1}}\\ \cdots &{} \cdots &{} \cdots &{} \cdots \\ {y{'_{m - k}}}&{}{y{'_{m - k + 1}}}&{} \cdots &{}{y{'_{m - 1}}} \end{array}} \right] $$, and the test set predicted output is column vector ${[y{'_1},y{'_2}, \cdots y{'_m}]^T}$. The LSTM neural network model is trained using the training set and validation set, that is, the model is trained to predict the next time step data of the reconstruction target data time series using the available data of *k* time steps. Then, using the closed-loop prediction of LSTM, the previously predicted values are iteratively passed to the next step prediction as input to the LSTM network to complete the iterative prediction of the data. This prediction process does not require input of any other data except the original data required for the first prediction. The test set output column vector is the data reconstruction result obtained by LSTM autocorrelation repair.
4. Analyze the error between predicted value ${[y{'_1},y{'_2}, \cdots y{'_m}]^T}$ and missing value ${[{y_1},{y_2}, \cdots {y_m}]^T}$, and evaluate the performance of model missing data repair.

### Data correlation analysis

With the rapid development of modern big data disciplines, research findings regarding time series data have been widely used in the finance, machinery, medical and construction industries. As typical time series data, structural monitoring data usually have significant or implicit correlations at each measuring point. Mining these correlations can efficiently reuse data and address missing data problems. At present, research on data correlation is mainly carried out by mathematical methods. In this paper, the Pearson correlation coefficient method is selected as the main method of correlation analysis for the correlation characteristics of monitoring data.

The Pearson correlation coefficient, also known as the product moment correlation coefficient, was first proposed by Karl Pearson^45^. It is mainly used to analyse the linear correlation between time series data and is widely used in signal analysis, fault diagnosis, numerical prediction and other fields. For time series data *X* and *Y*, the Pearson correlation coefficient formula is shown in Eq (1);

1 $$\begin{aligned} P = \frac{{\sum \limits _{i = 1}^n {({X_i} - {\bar{X}})({Y_i} - {\bar{Y}})} }}{{\sqrt{\sum \limits _{i = 1}^n {{{({X_i} - {\bar{X}})}^2}} } \sqrt{\sum \limits _{i = 1}^n {{{({Y_i} - {\bar{Y}})}^2}} } }} \end{aligned}$$

*P* ranges from $-1$ to 1. The closer the absolute value is to 1, the higher the linear correlation between *X* and *Y* is. The closer the absolute value is to 0, the lower the linear correlation between *X* and *Y* is. When the correlation coefficient is greater than 0, the two sets of sequence data are positively correlated; conversely, they are negatively correlated. The evaluation criteria of the Pearson coefficient are shown in Table 1.

**Table 1:** Pearson correlation coefficient evaluation criteria.

Coefficient *P*	Degree of relatedness
$0.8\le |P|\le 1$	high correlation
$0.5\le |P|\le 0.8$	moderate correlation
$0.3\le |P|\le 0.5$	low correlation
$0\le |P|\le 0.3$	Very weak or unrelated

### LSTM neural network

**Figure 2:**
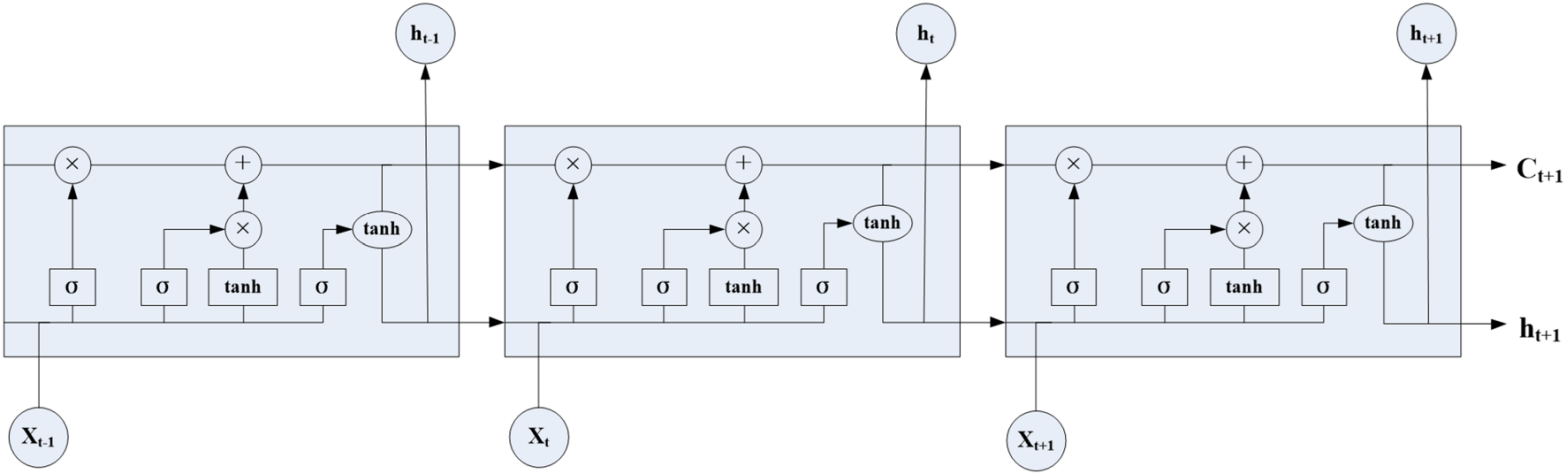
LSTM network structure.

The LSTM neural network is a deep learning technique based on the recurrent neural network (RNN). RNN is commonly employed for processing and predicting sequence data. It has been applied in various domains, such as speech recognition, text classification, machine translation, and image analysis. The key feature of RNN is its ability to receive information from other neurons and feed its own outputs back into neurons, making it suitable for handling time series data. Additionally, the shared parameters and repetitive structure of RNN reduce the number of required training parameters and enable the model to handle data of different lengths. However, pure RNN suffers from issues such as gradient explosion or vanishing gradients, impeding the long-term data characteristic extraction and hindering training convergence. The LSTM neural network addresses these challenges by incorporating LSTM units with input, output, and forget gates to regulate the flow of information. This enables LSTM to adjust its focus on relevant sequence data and encode the entire string, making it more effective in analysing and processing longer sequences. Fig. 2 illustrates the basic network structure of LSTM, with each unit performing computations based on a specific formula (2).

2 $$\begin{aligned} \left\{ \begin{aligned} {i_t}&= \sigma ({W_{ii}}{x_t} + {b_{ii}} + {W_{hi}}{h_{t - 1}} + {b_{hi}})\\ {f_t}&= \sigma ({W_{if}}{x_t} + {b_{if}} + {W_{hf}}{h_{t - 1}} + {b_{hf}})\\ {g_t}&= tanh({W_{ig}}{x_t} + {b_{ig}} + {W_{hg}}{h_{t - 1}} + {b_{hg}})\\ {o_t}&= \sigma ({W_{i0}}{x_t} + {b_{i0}} + {W_{h0}}{h_{t - 1}} + {b_{h0}})\\ {c_t}&= {f_t} \times {c_{t - 1}} + {i_t} \times {g_t}\\ {h_t}&= {o_t} \times \tanh ({c_t}) \end{aligned} \right. \end{aligned}$$

where $c_t$ represents the tuple state at time *t*, with an initial hidden state of 0; $i_t$ refers to the input gate; $f_t$ represents the forget gate; $g_t$ corresponds to the selection gate; and $o_t$ represents the output gate.

The LSTM model for missing data imputation in the SHM system is composed of four LSTM memory units and a fully connected layer, as shown in the LSTM model part of Fig. 1. The input layer inputs the standardized data set into the LSTM model. The hidden layer is composed of LSTM units, where the input data sequence is processed by the dropout operation through LSTM units to prevent overfitting and the output of the LSTM units is then mapped to the output layer^46^. The output layer denormalizes the output results of the previous layer to obtain the monitoring data corresponding to each moment of the input data set. During model training, the Adam optimization algorithm with excellent performance is used to minimize the loss function, and the model parameters are updated through iteration until the optimal network structure is obtained. Data recovery is defined as using existing temporal data or highly correlated adjacent point data to predict the monitoring data of missing points. For example, when the input test set is a monitoring data sequence of $\left\{ {{x_1},{x_2}, \cdot \cdot \cdot ,{x_{t - 1}},{x_t}} \right\}$ , ${x_{t + 1}}$ is the missing data at the next moment. Then, the monitoring data of $x{'_{t + 1}}$ can be obtained by the following formula (3):

3 $$\begin{aligned} x{'_{t + 1}} = W\cdot {h_t} + b \end{aligned}$$

where *w* is the weight connecting the LSTM and the output layer, $h_t$ can be calculated by formula, and *b* is the deviation of the output layer. Finally, the predicted missing monitoring data ${x_{t + 1}}$ can be obtained from $x{'_{t + 1}}$ by anti-standardization.

### Model evaluation criteria

In this section, the evaluation criteria for the results of the model in repairing missing data are explained. Root mean square error (RMSE) and mean absolute percent error (MAPE) are used as performance indicators to assess the ’performance and data repair effectiveness of the model.

RMSE calculates the square root of the sum of squared differences between the predicted values and the true values, divided by the number of data points, as shown in formula (4). A smaller RMSE value indicates better effectiveness in repairing missing monitoring data, while a larger value suggests poorer data repair effectiveness.

MAPE calculates the absolute sum of the differences between the predicted values and the true values, divided by the true values and then multiplied by 100, as shown in formula (5). Similar to the evaluation criterion of RMSE, a smaller MAPE value indicates better data repair performance^47^.

4 $$\begin{aligned} RMSE = \sqrt{\frac{1}{N}\sum \limits _{i = 1}^N {{{\left( {{x_i} -\hat{x}_{i} } \right) }^2}} } \end{aligned}$$

5 $$\begin{aligned} MAPE = \frac{1}{N}\sum \limits _{i = 1}^N {\left| {\frac{{{x_i} - \hat{x}_{i}}}{{{x_i}}}} \right| } \times 100\% \end{aligned}$$

In the above two formulas, ${x_i}$ is the *i*-th true data, $\hat{x}_{i}$ is the *i*-th predicted data, and *N* is the length of the data.

## Research on monitoring data recovery of grid construction

### Overview of grid jacking project

**Figure 3:**
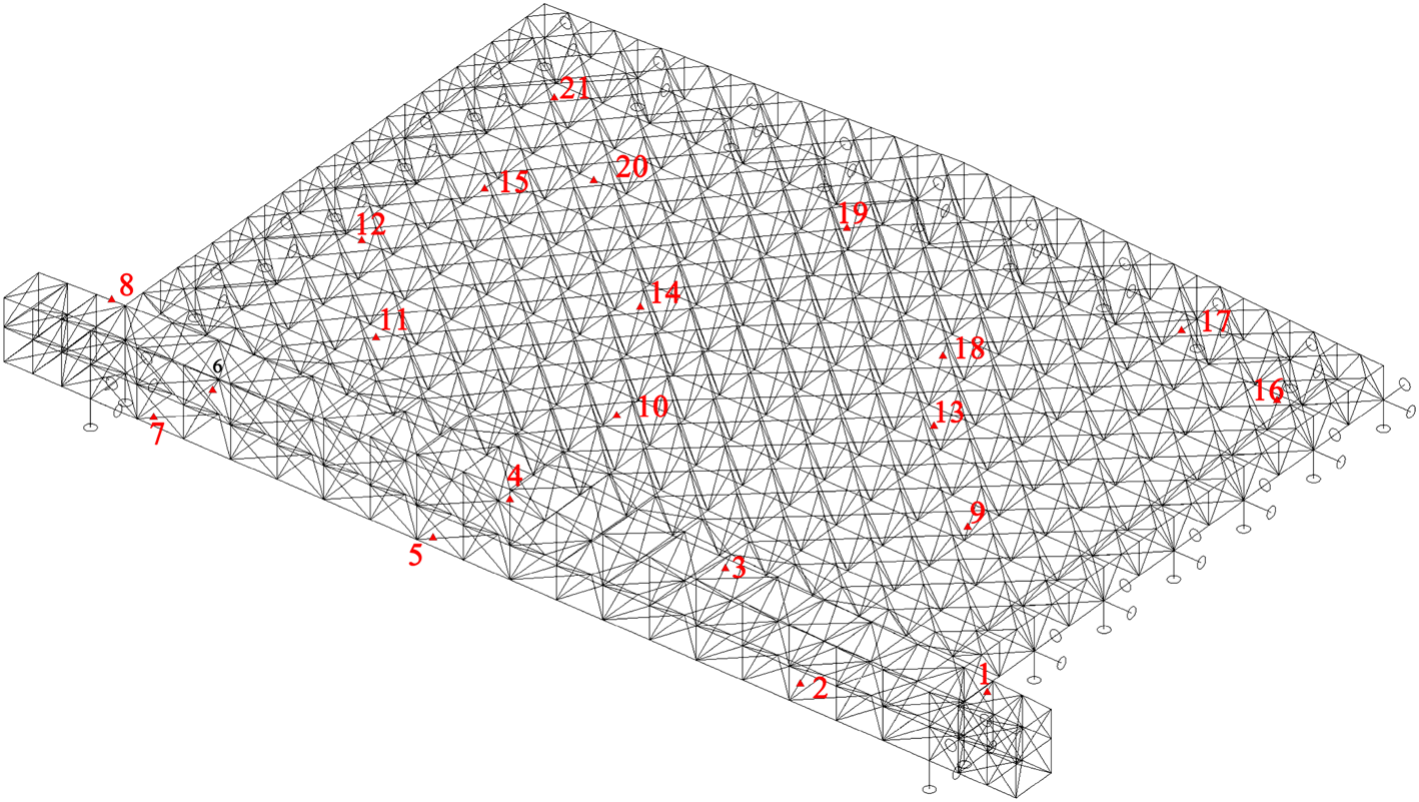
Orthogonal inclined grid structure.

**Figure 4:**
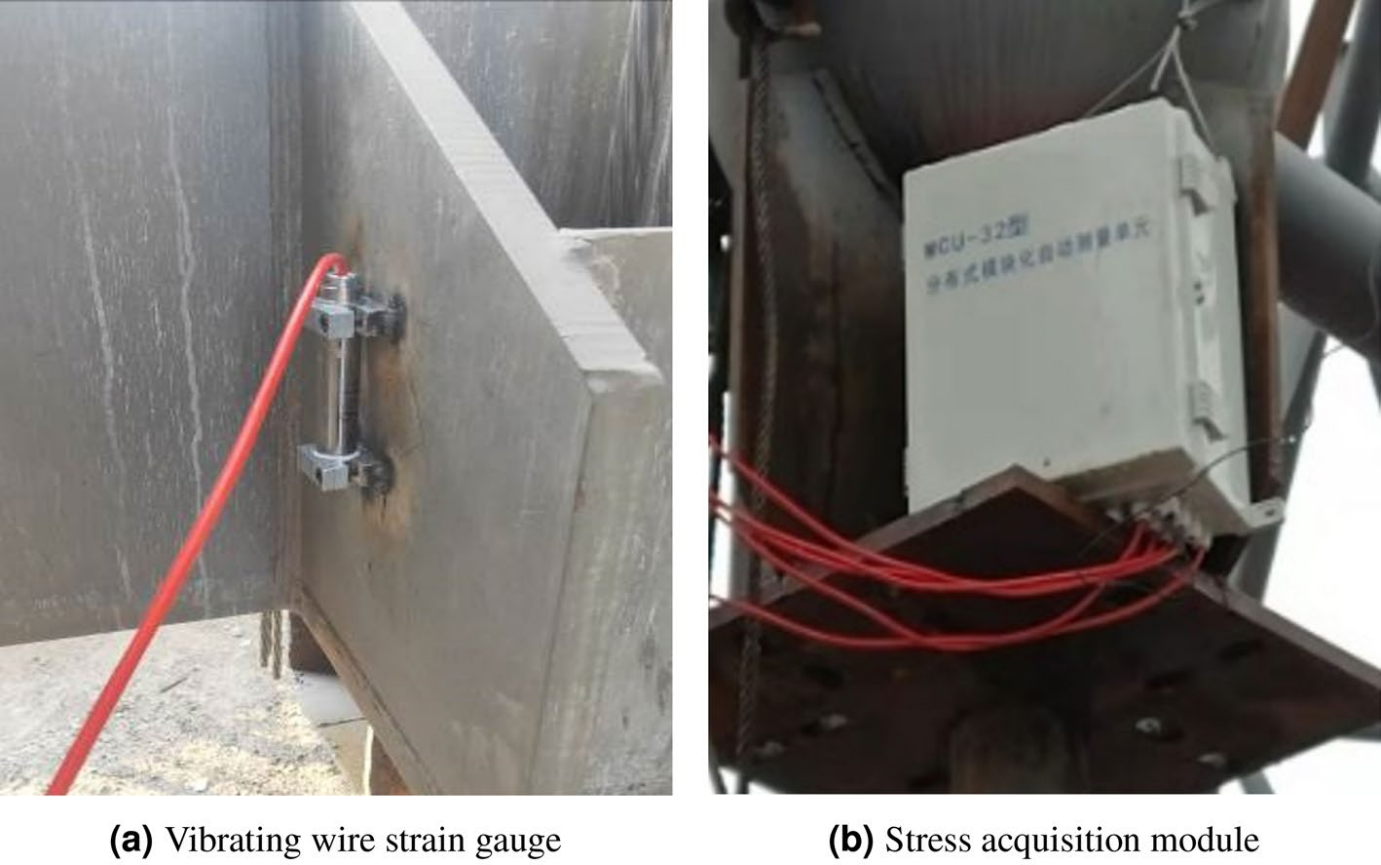
Equipment layout details.

This section investigates on the applicability and robustness of structural response repair methods using stress monitoring data from a steel structural truss roof system with overall lifting construction. The structure under investigation is a maintenance workshop, utilizing a large-span steel structure to ensure sufficient working space. This structure consists of two layers of orthogonal inclined trusses, with three sides supported and one side open, spanning 93 m in width and 65 m in length. The lower chord is supported, and the main grid size is 5.17 m $\times$ 5 m, with a slope of 5% and a maximum truss thickness of 5.925 m at the highest point. The lower chord is located at an elevation of 21.60 m, and the nodes are welded hollow sphere nodes. The truss at the open side adopts two sets of three-layer tubular trusses, with a maximum truss thickness of 9.525 m and a lower chord centre elevation of 18.00 m. A structural monitoring system consisting of 21 vibrating wire stress sensors, 18 displacement measurement points, and 4 data acquisition boxes was established at the beginning of construction to capture the internal force evolution and performance changes during the lifting stage. The overall truss structure and stress measurement points are illustrated in Fig. 3. Stress sensors are distributed on the upper chord, web members, and lower chord. The on-site stress monitoring equipment is shown in Fig. 4a,b, and the specific parameters of the stress monitoring devices are listed in Table 2.

**Table 2:** Stress equipment parameters.

Parameter	Numerical value
measuring scale distance	100 mm
effective diameter	22 mm
End diameter	24 mm
Tensile strain measurement range	800 $\mu \varepsilon$
Compressive strain measurement range	1200 $\mu \varepsilon$
Temperature measurement range	$-25 \sim +60^{\circ }\textrm{C}$
temperature measurement precision	$\pm 0.5^{\circ }\textrm{C}$
temperature correction coefficient	$\approx 13.5\times 10^{-6} / ^{\circ }\textrm{C}$
elastic modulus	300$\sim$500 Mpa
insulation resistance	$\ge$50 M$\Omega$

### Data selection and model training

**Figure 5:**
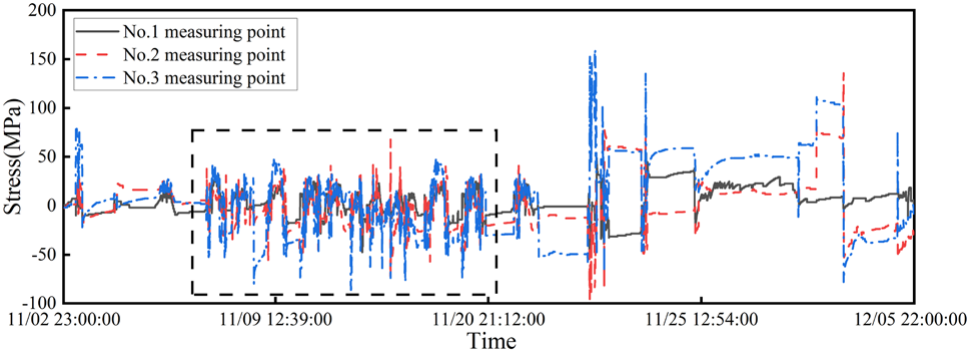
Stress data of measuring points.

Due to the large volume of data in this study, stress data from measurement point 1 during the period from November 7, 2020 to November 20, 2020 is selected as the target for the missing data investigation, as indicated by the dashed box in Fig. 5. For the data set used in the damage identification method considering spatial correlation among multiple measurement points, the correlation between measurement points *b* and *c* with measurement point a was calculated using the Pearson correlation coefficient. These results are presented in Table 3. Table 3 shows that for the repair of spatial correlation among multiple measurement points, the results of measurement point 2 were chosen as a highly correlated data sequence to construct the training data set for the missing data of measurement point 1.

**Table 3:** Pearson coefficient results.

Measuring point number	Pearson correlation coefficient
2$^{\textrm{nd}}$	0.812
3$^{\textrm{nd}}$	0.451

**Figure 6:**
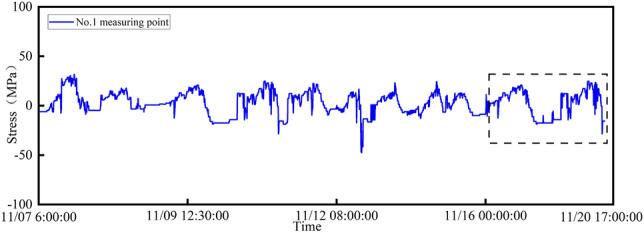
Stress data of No.12 measuring point.

This study aimed to monitor the stress response of the monitoring system from 6:00 on November 7, 2020 to 17:00 on November 20, 2020. The first of the data were set as the training set, the middle 10% of the data were set as the validation set, and the last 20% were set as the missing test set. The test set is the part selected by the virtual box in Figure 6. The hardware configuration and software environment used for LSTM model construction are shown in Table 4.

**Table 4:** Hardware configuration and software environment.

Experimental environment	Configuration details
CPU model	Core i5-10400 @ 2.90 GHz
GPU model	Nvidia GeForce GTX 1060
Memory	16G
system environment	Win10 64bit
programming language	Python3.7
programming language	Python3.7
Keras and TensorFlow version	2.3.1/2.9.1

#### Analysis of missing data imputation results

**Figure 7:**
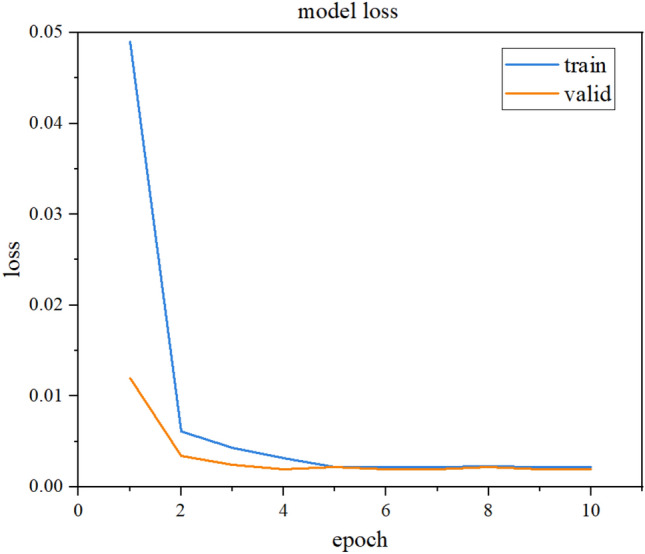
Damage curve of self-correlation LSTM model.

**Figure 8:**
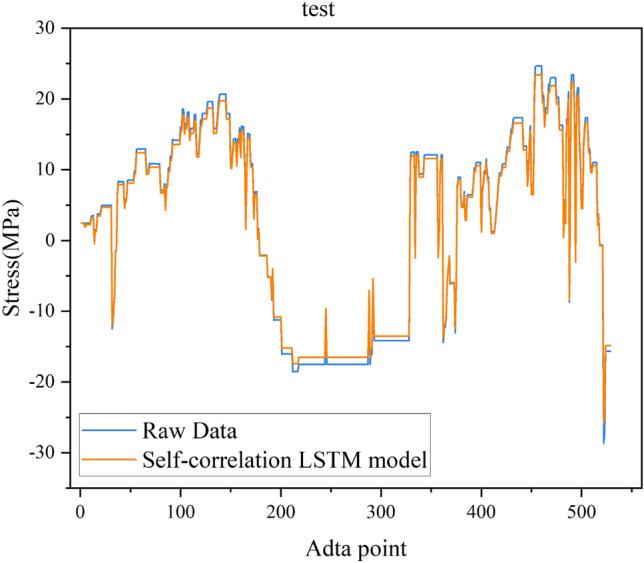
Self-correlation LSTM model test fitting results.

Fig. 7 shows the LSTM model training loss curve for the single test point autocorrelation data repair method. The figure clearly shows that the LSTM loss value decreases continuously as the model iterates and converges when the model iterates times, with a final loss value of approximately 0.002. The overlap of the training and testing loss curves indicates that the LSTM model also performs well on the test set; that is, the model has good generalization ability. Fig. 8 visualizes the model test set fitting results, where blue represents the original monitoring data and yellow represents the fitting results. This figure shows that most of the LSTM model fitting results overlap with the original data and the monitoring data trend is consistent with that of the actual monitoring results. The error mainly occurs at the peak of the signal when the signal changes sharply, and the fitting results are generally 1.2 Mpa smaller than the true value. The stress monitoring value predicted by LSTM is consistent with the actual monitoring result trend, and the overall value is close.

**Figure 9:**
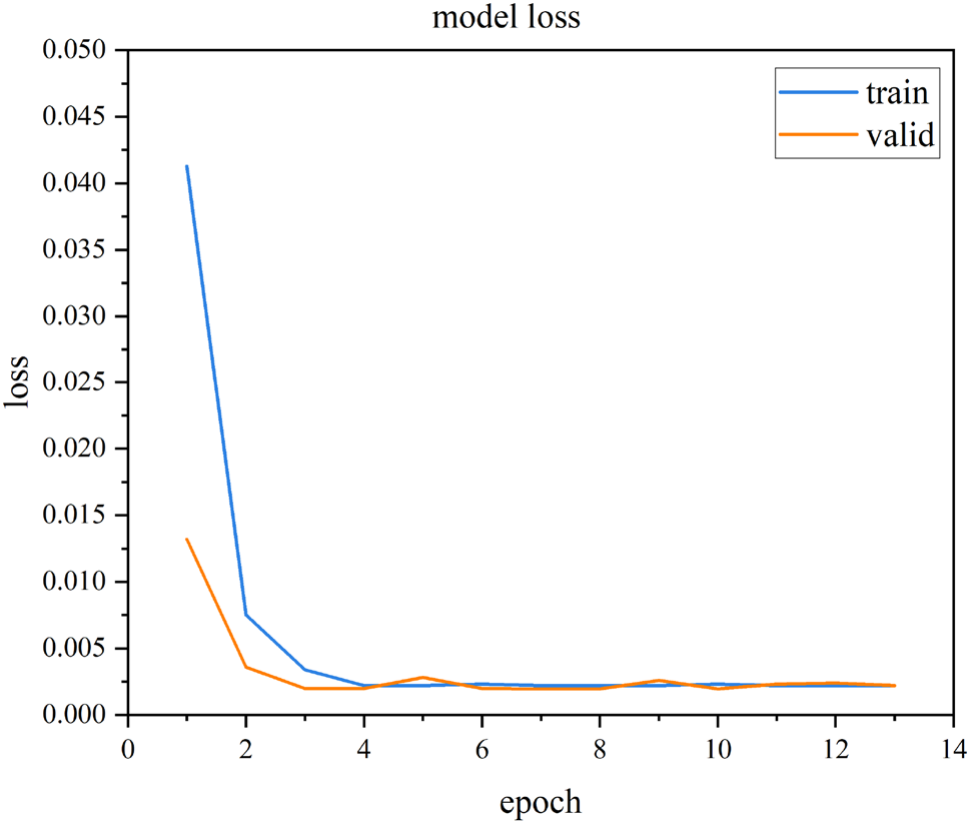
Damage curve of high correlation LSTM model.

**Figure 10:**
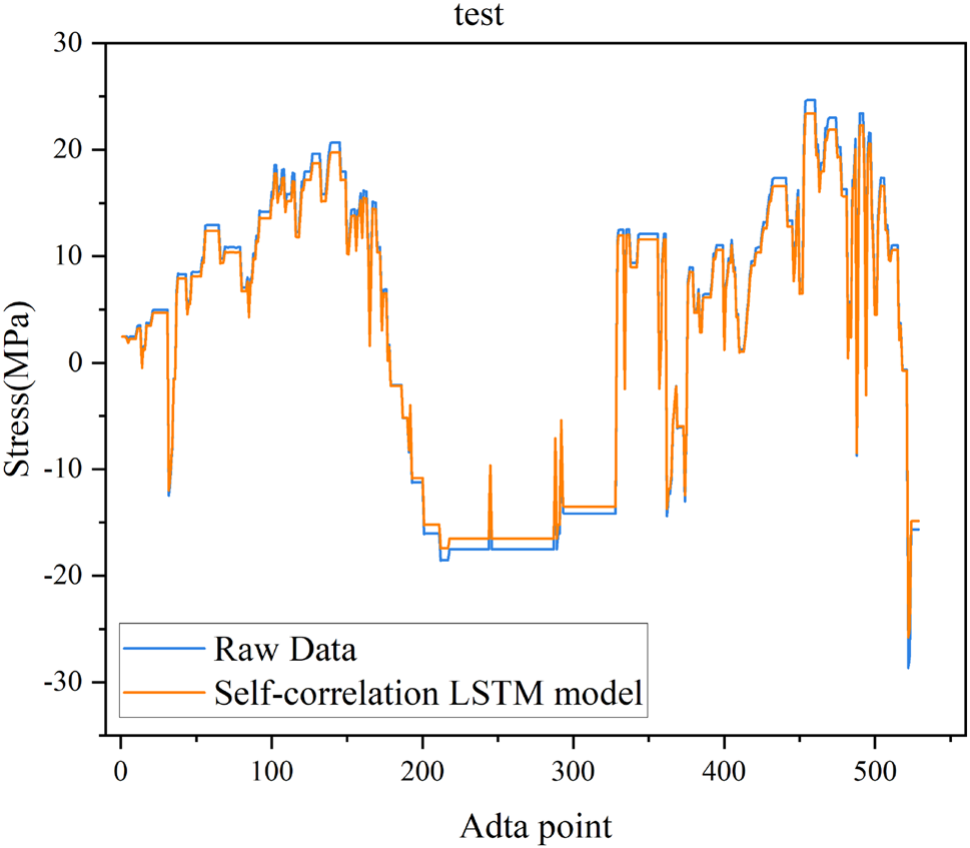
High correlation LSTM model test fitting results.

The multitest point spatial high correlation data repair method uses the complete data of test point 2 during the missing period of test point 1 to train the LSTM model. The loss curve is shown in Fig. 9. Similar to the single test point, the LSTM loss value decreases continuously as the number of iterations increases, converges when the number of iterations reaches 20, and then remains unchanged in subsequent iterations. Fig. 10 shows the model test set fitting results, where blue represents the original monitoring data and yellow represents the fitting results. The figure shows that the LSTM model predicts the missing results of test point 1 using the spatially highly correlated test point 2 more accurately than the autocorrelation model. This indicates that using the spatial correlation of multiple test points can improve the ability of the LSTM model to repair structural health monitoring data.

### Effects of different missing rates on the model

**Figure 11:**
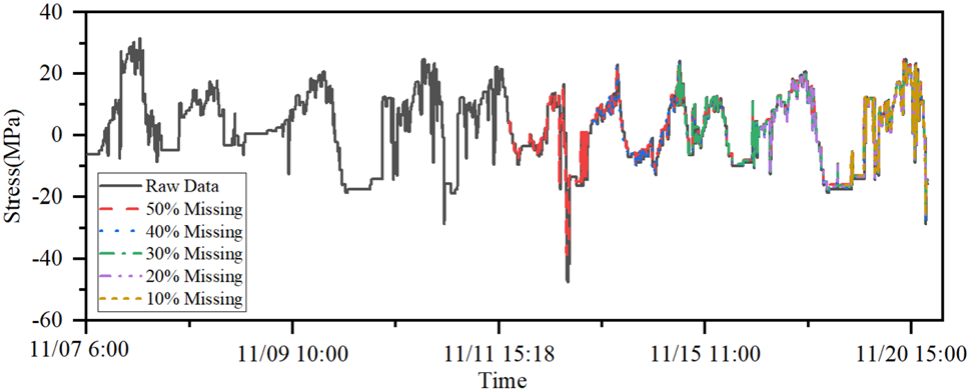
Different loss rate results.

Since the amount of data damage in actual engineering is uncertain, the stress data of test point 1 is still taken as the research object in this section. Five different data loss ratios of 10%, 20%, 30%, 40% and 50% are set to analyse the applicability range of the proposed LSTM-based data repair method. The model training process is not repeated, and the training data amount and the missing data segment are different. The results of this process are shown in Fig. 11. To further illustrate the effect of different loss rates on the data repair performance of the model, RMSE and MAPE indicators are used. These results are shown in Table 5.

**Table 5:** Comparison of prediction model performance index.

Proportion of data loss	RMSE	MAPE
10%	0.681	1.91%
20%	0.695	1.50%
30%	1.553	4.26%
40%	1.915	8.95%
50%	2.214	14.87%

Figure 11 illustrates the outcomes of repairing the model concerning stress data at test point 1 under varying loss ratios. Signals characterized by 10% to 30% data loss exhibit a robust consistency with the original signals post-recovery. Within this specific range of data loss ratios, the recuperated signals almost perfectly align with the originals. The signal recovered from a 50% loss ratio displays a waveform pattern strikingly similar to the original, albeit with minor amplitude variations at certain points that remain within an acceptable range.

In Table 5, the quantification of recovery errors is presented through performance evaluation metrics. The findings indicate that, with an escalation in the severity of data loss, the recovery error experiences a gradual increase. This implies that as the data loss ratio rises, the disparity between the original signal and the recovered signal also progressively widens.

### Comparison of data missing repair models

**Figure 12:**
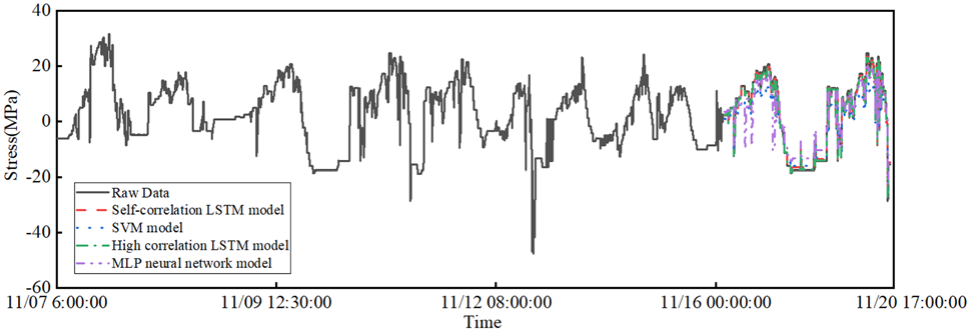
Overall repair results of model data.

**Figure 13:**
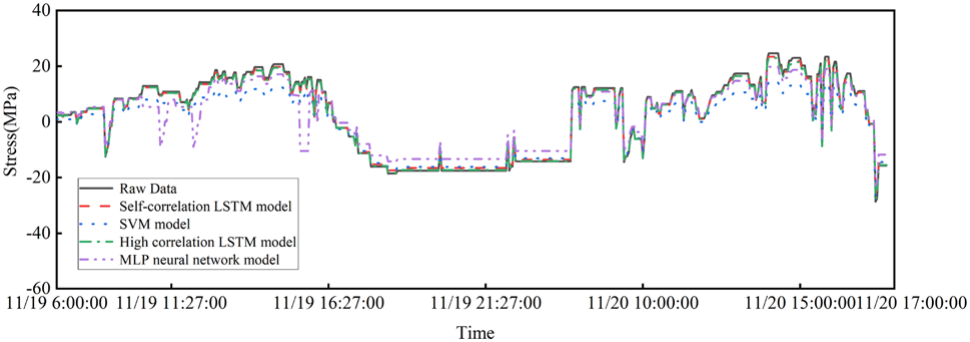
Model data missing segment repair results.

To compare the performance of the LSTM model in monitoring data repair, two different traditional prediction models, namely, support vector machine and MLP neural network, are selected to repair the data of test point 1. The support vector machine model uses a radial basis kernel function with penalty parameter $c=0.5$ and tolerance error parameter 0.01. The MLP neural network has 6 neurons in the hidden layer and uses Tansig as the activation function. The results are shown in Figs. 12 and 13. In this section, RMSE and MAPE are used to further illustrate the data repair performance of the structure monitoring missing data. These results are shown in Table 6. The RMSE and MAPE indicators measure the error between the predicted stress data and the actual stress data. The smaller the value is, the closer the data repair result is to the real data. Figs. 12 and 13 show the missing data repair results of test point 1 by four different data repair models. The figures show that the two LSTM-based data repair models have better effects than the other models. The data repair model based on the support vector machine fits the trend of missing data well and can reflect the overall change in stress data in the missing segment, but the prediction result of specific missing values is poor, with a general difference of 5 Mpa. The repair based on the MLP neural network predicts the specific missing values close to the original data well but fits the overall trend of stress data poorly, showing three obvious stress drop segments that do not match the actual working conditions at 10:00-12:00 and 16:00 on November 19.

**Table 6:** Prediction model performance index comparison.

Data repair method	RMSE	MAPE
Autocorrelation LSTM model	0.6802	1.499%
SVM model	4.3042	23.551%
High correlation LSTM model	0.6015	2.720%
MLP model	4.6174	4.818%

As shown in Table 6, among the four missing data repair models, the two prediction repair methods using the LSTM model have significantly better accuracy than the support vector machine and MLP neural network, with RMSE reduced by more than 3.5 and MAPE reduced by 20%. This indicates that the long short-term memory neural network performs better and can mine better temporal data features than traditional machine learning algorithms in data prediction, making it suitable for missing data repair research on structural health monitoring. Although the two LSTM data repair models have excellent performance, the LSTM model based on spatial high correlation has slightly higher repair accuracy than the repair method based on stress data self-correlation.

## Operation data imputation of Terminal *F*-hall

### Project overview

**Figure 14:**
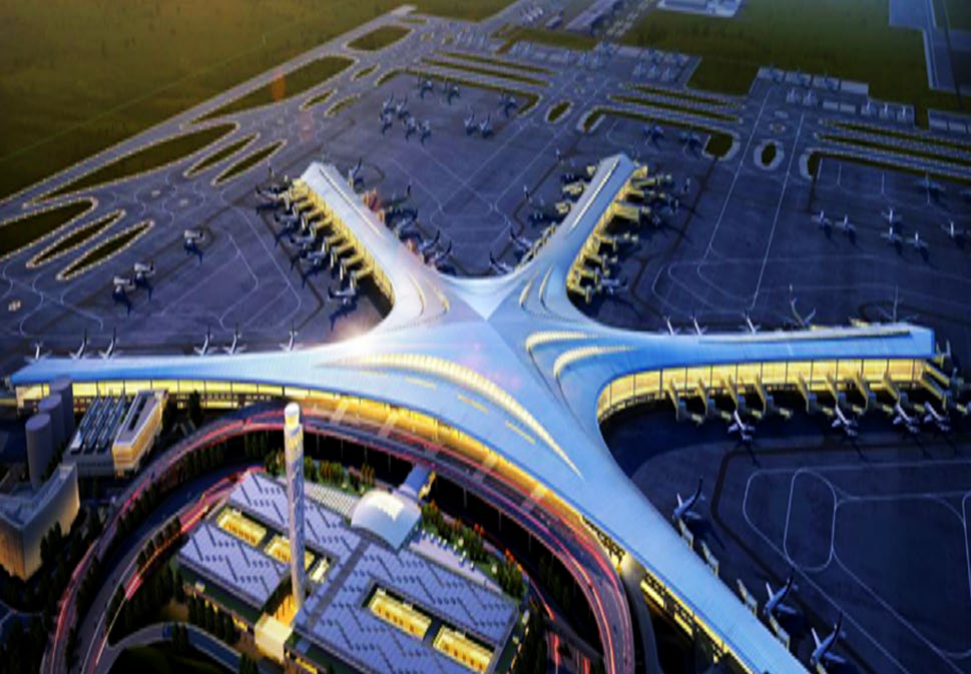
Effect diagram of Terminal building, Qingdao Jiao-dong International Airport.

**Figure 15:**
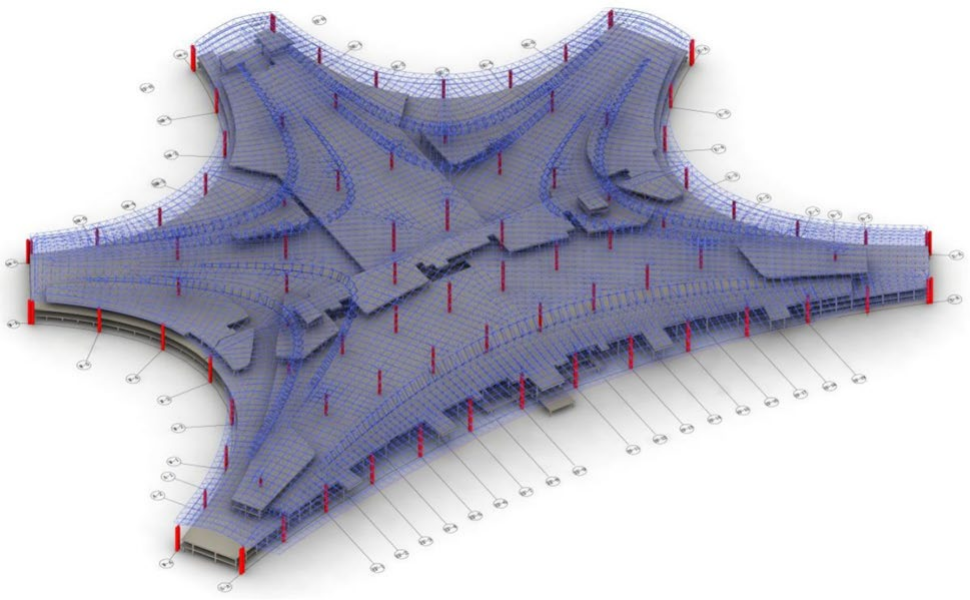
Axonometric drawing of *F* Hall structure.

**Figure 16:**
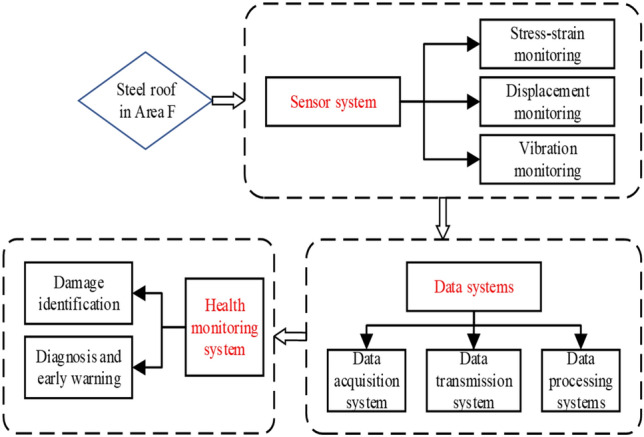
Composition of structural health monitoring system.

**Figure 17:**
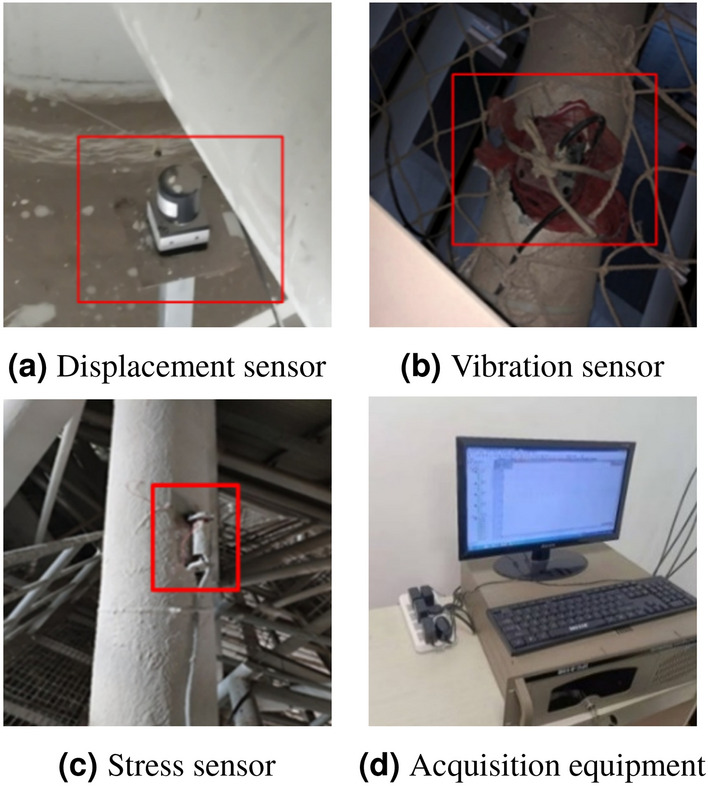
Site layout of structural health monitoring system.

In addition to stress monitoring during construction, most structural health monitoring systems involve various types of monitoring data, such as displacement and vibration, and different structural response data face the same data loss problem. Therefore, the monitoring system of Qingdao Jiao-dong International Airport Terminal *F* is taken as the research object in this section. Missing data repair research is conducted on stress, displacement and vibration data based on LSTM. The terminal building of Qingdao Jiao-dong International Airport has a “starfish” layout. The steel roof of Terminal *F* has a plan size of 500 metres $\times$ 339 metres, and the highest point of the metal roof is 42.150 metres. The terminal rendering and hall axonometric diagram are shown in Figs. 14 and 15. A structural health monitoring system consisting of 55 strain sensors, 18 level gauges, 16 wire displacement sensors, 14 acceleration sensors, 4 acquisition boxes and data analysis modules is established to ensure structural safety. The specific composition of this system is shown in Fig. 16, and the field measurement points are shown in Fig. 17.

### Data selection and model training

**Figure 18:**
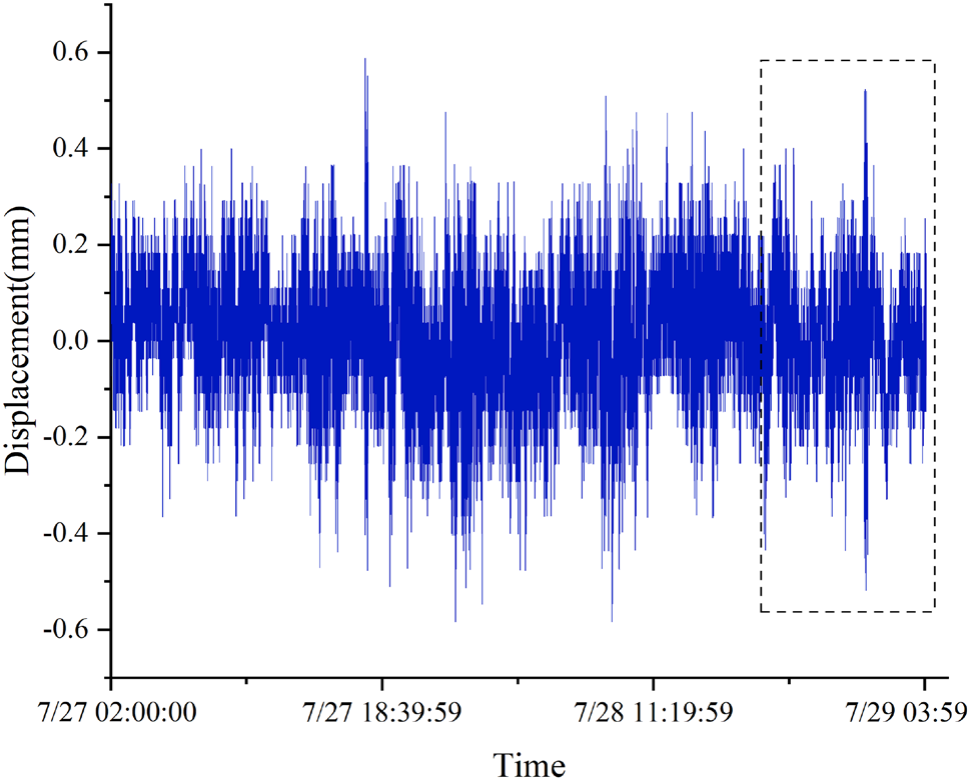
Displacement data (sampling frequency 1 Hz).

**Figure 19:**
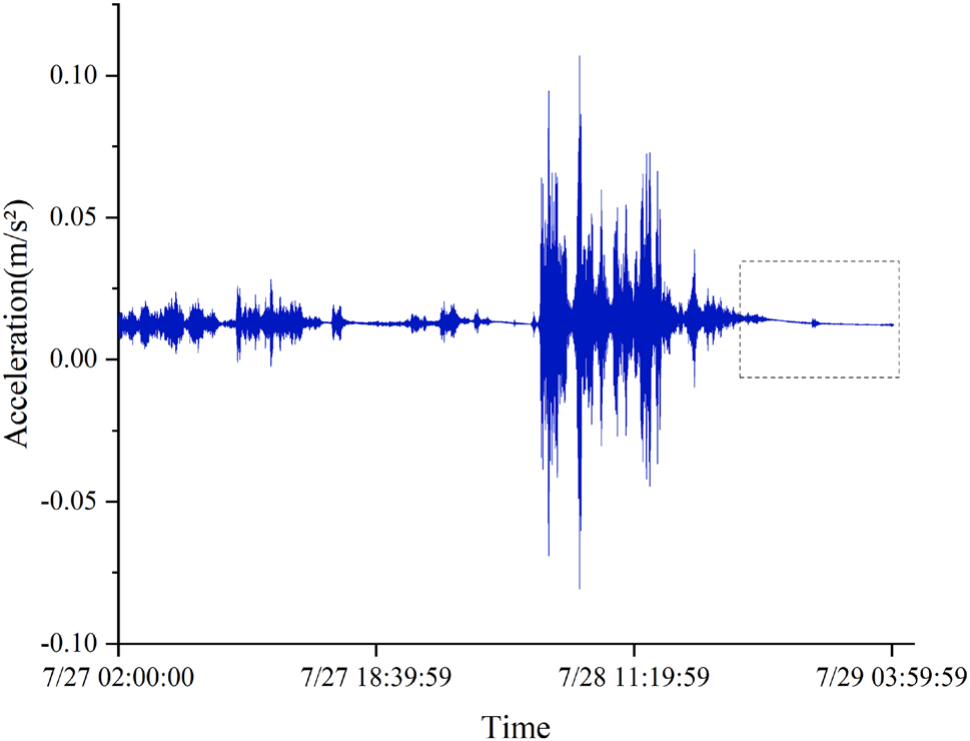
Acceleration data (sampling frequency 1 Hz).

**Figure 20:**
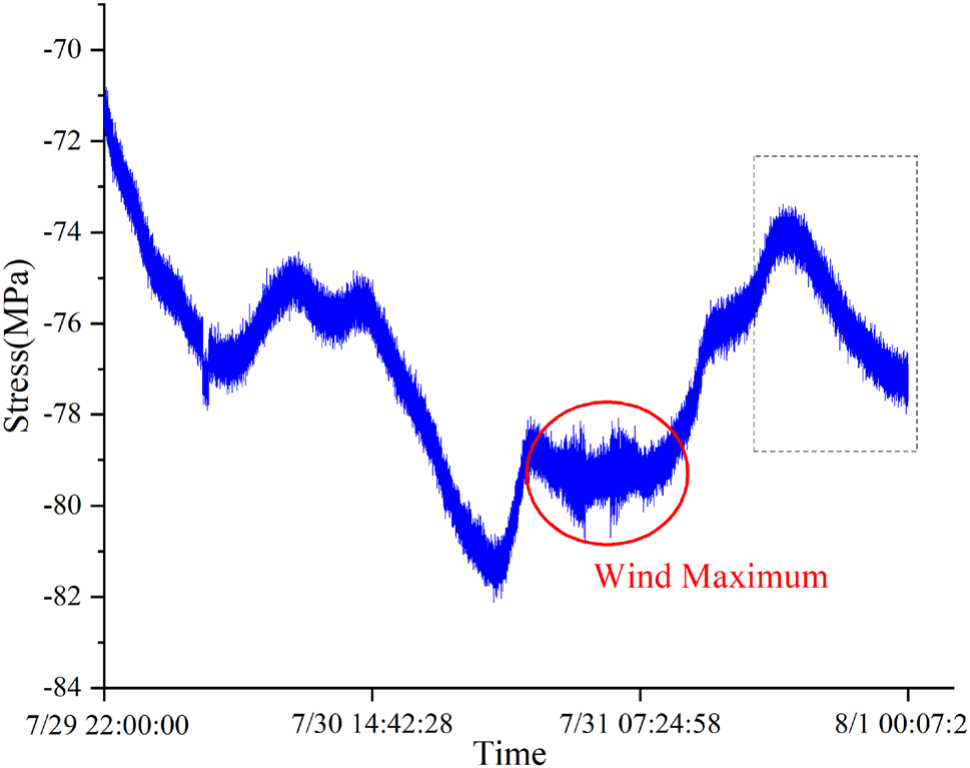
Stress data (sampling frequency 0.5 Hz).

In real structural engineering, daily monitoring data are stable, and the impact of missing data segments on structural safety assessment and analysis is usually limited, so repairing these segments is not very meaningful. Therefore, the structural monitoring data of the terminal building from 2:00 on July 27, 2021 to 4:00 on July 29, 2021, when the typhoon “Fireworks” crossed Shandong, is selected in this paper. The displacement, vibration, and stress data are shown in Figs. 18, 19, and 20.

**Figure 21:**
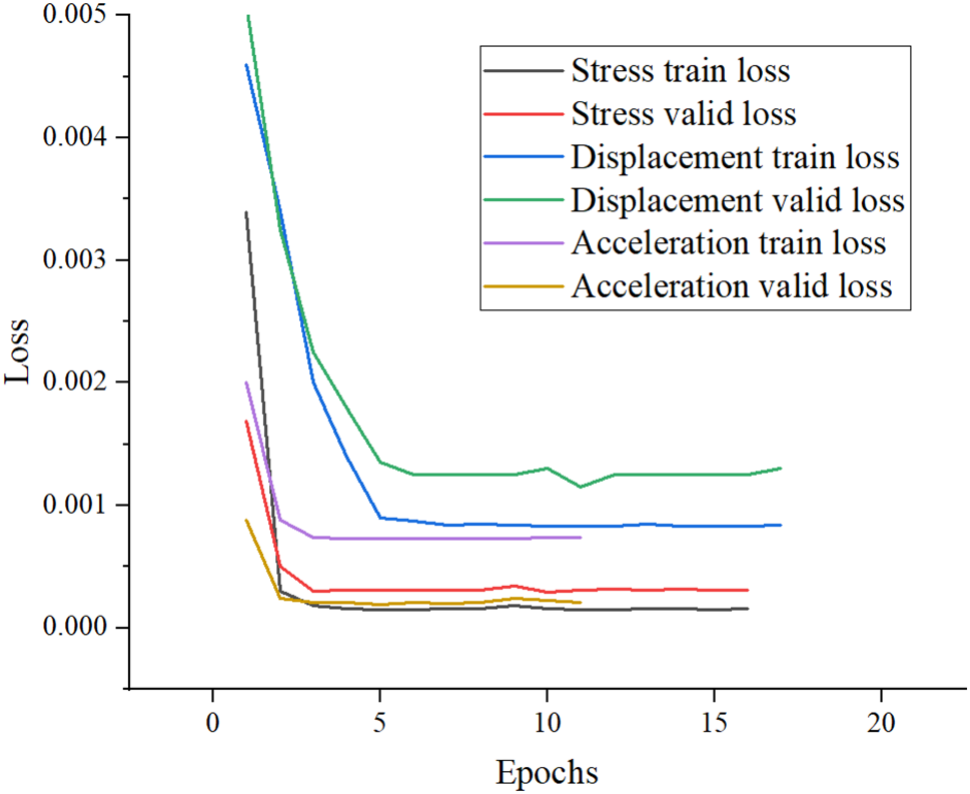
Training convergence curve.

For three different types of missing data, the LSTM model is still trained with a loss rate of 20%. The input size, LSTM structure, and training terminal condition are the same as those for the recovery of stress data from the overall lifting monitoring of the grid structure. The length of the training data set is 72000 (36000), and the length of the missing segment data set is 18000 (9000).

### Data Recovery Results

**Figure 22:**
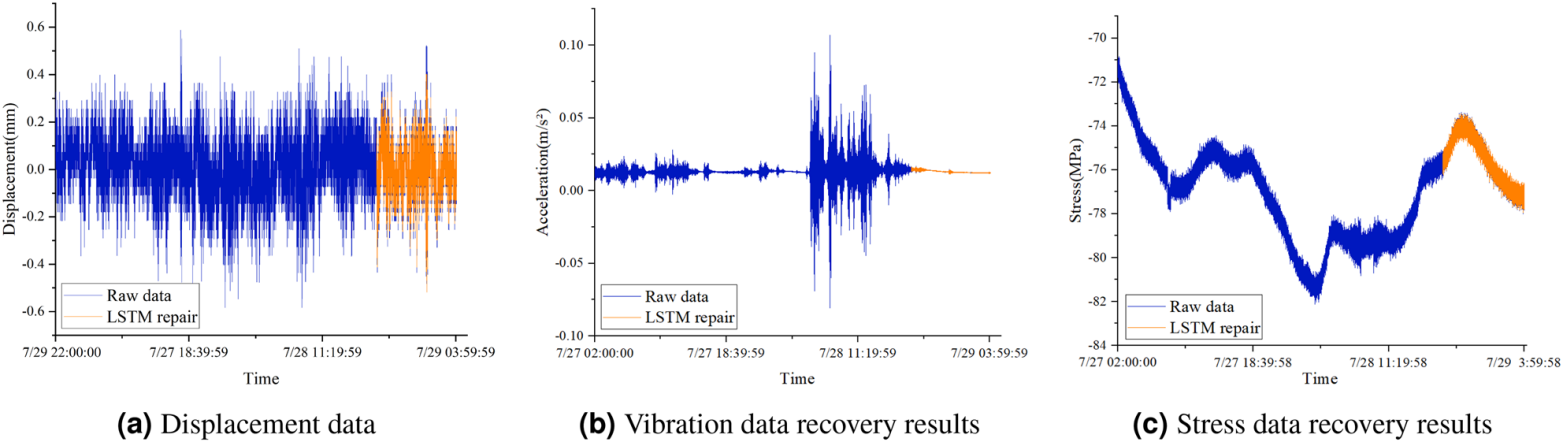
Recovery results of the missing segment.

Fig. 21 shows the training convergence curves of the displacement, vibration and stress data recovery models for the *F* hall of Qingdao Jiao-dong International Airport terminal building. The figure shows that the loss function decreases sharply in the initial stage of training and then converges. This indicates that the LSTM-based missing data recovery method can also fully train the LSTM model when facing displacement and vibration monitoring data. The data after 14:00 on July 31 are assumed missing. Fig. 22 depicts the recovery results of the missing segment, showing that the recovery curve has a small deviation from the original data. RMSE and MAPE indicators are used to evaluate the recovery of different types of data, and these results are shown in Table 7.

**Table 7:** Prediction model performance index comparison.

Data type	RMSE	MAPE
displacement	0.011428	5.4281%
vibration	9.326E-6	0.034392%
stress	0.018526	0.02192%

## Conclusion

Missing data in structural monitoring are an important factor in structural analysis reliability and influence the safety assessment results of buildings. In this paper, two LSTM-based missing data recovery methods for stress data missing in large-span spatial structure monitoring systems are proposed: one for isolated measurement points with self-correlation and one for multiple measurement points with high correlation. The data reconstruction effects of these two methods are discussed using the stress data from the lifting stage of the grid structure. The results show that both LSTM-based missing data recovery methods can accurately fit the stress variation law of the missing data segments, and the predicted stress results of these models are close to the real values, with an MSE of approximately 0.6 and MAPE below 15%. The LSTM recovery method using multiple measurement points with high correlation has slightly higher accuracy than the self-correlated LSTM data recovery method. In addition, the recovery method of the proposed LSTM model has much higher accuracy and smaller error than the prediction models of the support vector machine and MLP neural network in traditional machine learning algorithms.

## Discussion

This study explores the reconstruction of data based on LSTM neural networks and correlation analysis, focusing only on the stress or strain, acceleration, and displacement sensors in the construction and operation of the grid structure roof. No further algorithm optimization was performed. In the future, intelligent evolutionary algorithms can be introduced to optimize the hyperparameters of the LSTM neural network to achieve a lower error rate in data reconstruction. As the unit price of computing power continues to rise, more expensive algorithms relative to LSTM computing costs, such as generative adversarial networks and transformers, can also be applied to the data reconstruction process. More parameters may bring more reliable and accurate results.

## Data Availability

All data generated or analyzed during this study are included in this published article.
